# Patient-level reclassification and discordance between SOFA and SOFA-2: a multicenter ICU study

**DOI:** 10.1186/s13054-026-06241-0

**Published:** 2026-08-11

**Authors:** Shohei Ono, Shigehiko Uchino, Yutaka Kumaido, Shunsuke Yawata, Shinshu Katayama

**Affiliations:** 1 Department of Anesthesiology and Critical Care Medicine, Jichi Medical University Saitama Medical Center, Saitama, Japan; 2 Department of Emergency and Critical Care Medicine, Jichi Medical University Saitama Medical Center, Saitama, Japan; 3 Graduate School of Data Science, Yokohama City University, Yokohama, Japan; 4 Division of Intensive Care, Department of Anesthesiology and Intensive Care Medicine, Jichi Medical University, Tochigi, Japan; 5 Jichi Medical University Saitama Medical Center, 1-847 Amanuma- cho, Omiya-ku, Saitama, 330-8503 Japan

**Keywords:** SOFA score, SOFA-2, organ dysfunction, intensive care unit, reclassification

## Abstract

**Background:**

The Sequential Organ Failure Assessment (SOFA) score is widely used to quantify organ dysfunction in critically ill patients. A revised version, SOFA-2, was proposed to better reflect contemporary intensive care practice, but patient-level reclassification and the implications of differences between SOFA and SOFA-2 remain unclear.

**Methods:**

We conducted a retrospective multicenter cohort study using the OneICU database, a Japanese ICU registry including 15 hospitals. Adult ICU admissions between April 2012 and December 2025 were analyzed. Day-1 SOFA and SOFA-2 scores were calculated. Reclassification patterns were visualized using Sankey diagrams, and mortality patterns were assessed using heat maps. Score discordance was defined as SOFA deviation (dSOFA = SOFA-2 − SOFA) and categorized as Negative ( ≤ − 2), Minimal ( > − 2 and < 2), or Positive (≥ 2). Two primary exact-matching analyses were performed across dSOFA categories, separately matching patients on Day-1 SOFA-2 and conventional SOFA total scores.

**Results:**

Among 134,528 ICU admissions, SOFA-2 resulted in systematic patient-level reclassification. Upward shifts were most frequent in the brain, cardiovascular, and respiratory subscores, whereas the kidney subscore showed bidirectional changes. Mortality increased with higher SOFA-2 subscores at fixed SOFA and with higher SOFA subscores at fixed SOFA-2. After exact matching on SOFA-2, mortality was highest in the Negative dSOFA group, in which SOFA was higher than SOFA-2. Conversely, after exact matching on conventional SOFA, mortality was highest in the Positive dSOFA group, in which SOFA-2 was higher than SOFA.

**Conclusions:**

SOFA-2 introduces systematic reclassification at the patient level. Reciprocal exact-matching analyses demonstrated that patients assigned the same severity by one scoring system remained heterogeneous in mortality according to the other scoring system. SOFA and SOFA-2 may therefore provide overlapping but complementary information for assessing organ dysfunction severity.

**Supplementary Information:**

The online version contains supplementary material available at 10.1186/s13054-026-06241-0.

## Introduction

In 2025, an updated version of the Sequential Organ Failure Assessment score (SOFA-2) was proposed to better reflect contemporary intensive care practice [1, 2]. Although the SOFA score, introduced in 1996 [3], has long been a cornerstone for assessing organ dysfunction in critically ill patients [4–6], several components of the score no longer fully align with current therapeutic strategies and monitoring practices [7–10]. SOFA-2 introduces revisions to several organ subscores to better reflect modern therapeutic strategies and monitoring practices. In the original report [2], SOFA-2 demonstrated slightly improved discrimination for mortality compared with the conventional SOFA score (AUROC 0.79 vs. 0.77) across multiple large ICU databases [11–16], and subsequent external validation studies have reported consistent findings [17, 18].

Despite these encouraging results, key aspects of the transition from SOFA to SOFA-2 remain unclear. First, it is not well understood how individual patients are reclassified at the organ-subscore level when SOFA is replaced by SOFA-2, as previous reports have focused on aggregate score distributions rather than patient-level transitions. Second, the relationship between total SOFA and SOFA-2 scores has not been systematically evaluated, and the clinical implications of differences between the two scores remain uncertain.

To address these knowledge gaps, we analyzed a multicenter ICU database to investigate how organ dysfunction assessment changes when SOFA is replaced by SOFA-2. Specifically, we aimed to evaluate (1) patient-level reclassification of organ-specific subscores between SOFA and SOFA-2 and the clinical implications of these changes, and (2) the association between differences in total SOFA and SOFA-2 scores and patient mortality. We hypothesized that the transition to SOFA-2 would lead to systematic patient-level reclassification and that discordance between SOFA and SOFA-2 would be associated with mortality differences independent of baseline severity.

## Methods

### Design

We conducted a retrospective multicenter observational study using the OneICU database (provided by MeDiCU, Inc., Osaka, Japan, https://www.medicu.co.jp/), a Japanese ICU registry from 15 hospitals. The OneICU database records high-resolution ICU data at 1-minute intervals (vital signs, laboratory results, therapies, and clinical observations). The study was approved by the Institutional Review Board of Jichi Medical University Saitama Medical Center (S25-109) and conducted in accordance with ethical guidelines for de-identified routinely collected data, following STROBE [19].

### Data collection

The study period was from April 1, 2012 to December 31, 2025. From 164,455 ICU admissions in the database, we excluded patients < 18 years, ICU stay < 12 h, and admissions with missing ICU mortality. We extracted the following variables for each ICU admission: age, sex, body mass index, ICU readmission status, elective surgery status, APACHE III score, and Charlson Comorbidity Index. We also extracted use of key therapies and organ support modalities recorded in the database, including nasal high-flow oxygen, non-invasive positive pressure ventilation, invasive mechanical ventilation, veno-venous ECMO, veno-arterial ECMO, renal replacement therapy, vasoactive/inotropic agents (epinephrine, vasopressin, norepinephrine, dopamine, and dobutamine), and mechanical circulatory support (intra-aortic balloon pumping and microaxial flow pump). Outcomes included ICU mortality, in-hospital mortality, ICU length of stay, and hospital length of stay.

SOFA and SOFA-2 total scores were calculated at two temporal resolutions: Day-1 (first 24 h after ICU admission) and hourly. The detailed calculation rules for SOFA and SOFA-2 were prespecified and retrospectively applied to all eligible ICU admissions using routinely collected data in the OneICU database, as described in the Supplementary Methods. Score discordance between SOFA and SOFA-2 was quantified as SOFA deviation (dSOFA = SOFA-2 − SOFA) and categorized as Negative ( ≤ − 2), Minimal ( > − 2 and < 2), or Positive (≥ 2). The primary outcome was ICU mortality (death during the ICU stay).

### Statistical analysis

Continuous variables are reported as mean (SD) and categorical variables as n (%), compared using t-tests and chi-square tests, respectively. Reclassification was visualized using subscore-specific Sankey diagrams. To characterize prognostic implications of subscore-level reclassification, we created heat maps displaying observed ICU mortality (%) for each combination of SOFA and SOFA-2 organ subscores. We examined the distribution of SOFA deviation and ICU mortality across SOFA-2 severity strata. To compare ICU mortality at equivalent severity levels defined by each scoring system, we performed two primary exact-matching analyses across the three dSOFA categories [20]. Exact matching was used to construct comparison cohorts in which the prespecified matching characteristic—either the Day-1 SOFA-2 total score or the Day-1 SOFA total score—was identical across the dSOFA categories. Matching was conducted separately for each exact score value, thereby yielding identical distributions of the score used for matching across the three groups. Within each matched cohort, ICU mortality was compared across dSOFA categories within strata of the score used for matching. The conceptual framework for conditioning on either SOFA-2 or SOFA in the matched analyses was illustrated using a directed acyclic graph (DAG). As sensitivity analyses, we performed inverse probability weighting using multinomial propensity scores for the dSOFA categories and conducted a subgroup analysis restricted to patients with sepsis within the SOFA-2–matched cohort. Within the cohort matched on SOFA-2 total score, Day-1 organ subscores were summarized across deviation categories to identify which organ systems contributed most strongly to negative or positive deviations. All statistical tests were two-sided, and the threshold for statistical significance was set at α = 0.05. Data extraction and preprocessing were performed using Google BigQuery, and all statistical analyses were conducted using R version 4.5.2.

## Results

A total of 164,455 ICU admissions with 18,277,429 data points were available in the OneICU database (Supplementary Fig. 1). After excluding 11,563 admissions, the analytic cohort comprised 134,528 ICU admissions with 15,814,904 data points. ICU non-survivors were older (70.3 vs. 66.0 years), had higher APACHE III scores (149.0 vs. 82.4), more ICU readmissions (8.4% vs. 5.3%), fewer elective surgical admissions (3.2% vs. 14.7%), and higher baseline severity (SOFA 11.83 vs. 5.37; SOFA-2 11.86 vs. 5.52) than survivors (Table 1). The frequency of unmeasured variables for each SOFA and SOFA-2 component and the corresponding application of predefined score-assignment rules are summarized in Supplementary Table 1.

**Table 1:** Patient characteristics and outcomes

	All patients (*n* = 134528)	Survived (*n* = 128354)	Died in ICU (*n* = 6174)	*P*-value
Age, years, mean (sd)	66.2 (16.8)	66.0 (16.8)	70.3 (15.2)	<0.001
Female, n (%)	53,682 (39.9)	51,413 (40.1)	2269 (36.8)	< 0.001
Body Mass Index, kg/m2, mean (sd)	22.8 (6.1)	22.8 (6.2)	22.4 (4.9)	< 0.001
Readmission, n (%)	7275 (5.4)	6758 (5.3)	517 (8.4)	< 0.001
Elective Surgery, n (%)	19,001 (14.1)	18,804 (14.7)	197 (3.2)	< 0.001
APACHE III score, pts, mean (sd)	85.5 (39.9)	82.4 (37.4)	149.0 (37.5)	< 0.001
CCI, pts, mean (sd)	4.0 (3.0)	4.0 (3.0)	3.9 (2.7)	0.001
Treatment, n (%)				
Nasal High Flow	2872 (2.1)	2599 (2.0)	273 (4.4)	< 0.001
Non-Invasive Positive Pressure Ventilation	3024 (2.2)	2786 (2.2)	238 (3.9)	< 0.001
Invasive Mechanical Ventilation	36,680 (27.3)	32,296 (25.2)	4384 (71.0)	< 0.001
VV-ECMO	85 (0.1)	63 (0.0)	22 (0.4)	< 0.001
Epinephrine	1487 (1.1)	947 (0.7)	540 (8.7)	< 0.001
Vasopressin	4284 (3.2)	3032 (2.4)	1252 (20.3)	< 0.001
Norepinephrine	21,209 (15.8)	17,784 (13.9)	3425 (55.5)	< 0.001
Dopamine	9207 (6.8)	8015 (6.2)	1192 (19.3)	< 0.001
Dobutamine	8618 (6.4)	7666 (6.0)	952 (15.4)	< 0.001
Intra-Aortic Balloon Pumping	1089 (0.8)	889 (0.7)	200 (3.2)	< 0.001
Microaxial flow pump	131 (0.1)	87 (0.1)	44 (0.7)	< 0.001
VA-ECMO	924 (0.7)	517 (0.4)	407 (6.6)	< 0.001
Renal Replacement Therapy	5989 (4.5)	4642 (3.6)	1347 (21.8)	< 0.001
SOFA score, pts, mean (sd)	5.64 (4.12)	5.35 (3.87)	11.77 (4.39)	< 0.001
SOFA-2 score, pts, mean (sd)	5.81 (4.00)	5.52 (3.76)	11.86 (4.04)	< 0.001
Outcomes				
Hospital death, n (%)	10,145 (7.7)	3971 (3.2)	6174 (100.0)	< 0.001
Length of ICU Stay, days, mean (sd)	6.0 (10.0)	5.7 (8.8)	12.5 (22.8)	< 0.001
Length of Hospital Stay, days, mean (sd)	27.6 (34.5)	28.1 (34.7)	17.6 (29.5)	< 0.001

Categorical variables are presented as number (%); variables not labeled as n (%) are numerical and are presented as mean (SD). Abbreviations: APACHE III, Acute Physiology and Chronic Health Evaluation III; CCI, Charlson Comorbidity Index; IABP, Intra-Aorta Balloon Pumping; IMV, Invasive Mechanical Ventilation; NIPPV, Non-Invasive Positive Pressure Ventilation; RRT, Renal Replacement Therapy; VV-ECMO, Veno-Venous Extracorporeal Membrane Oxygenation; VA-ECMO, Veno-Arterial ExtraCorporeal Membrane Oxygenation

**Fig. 1:**
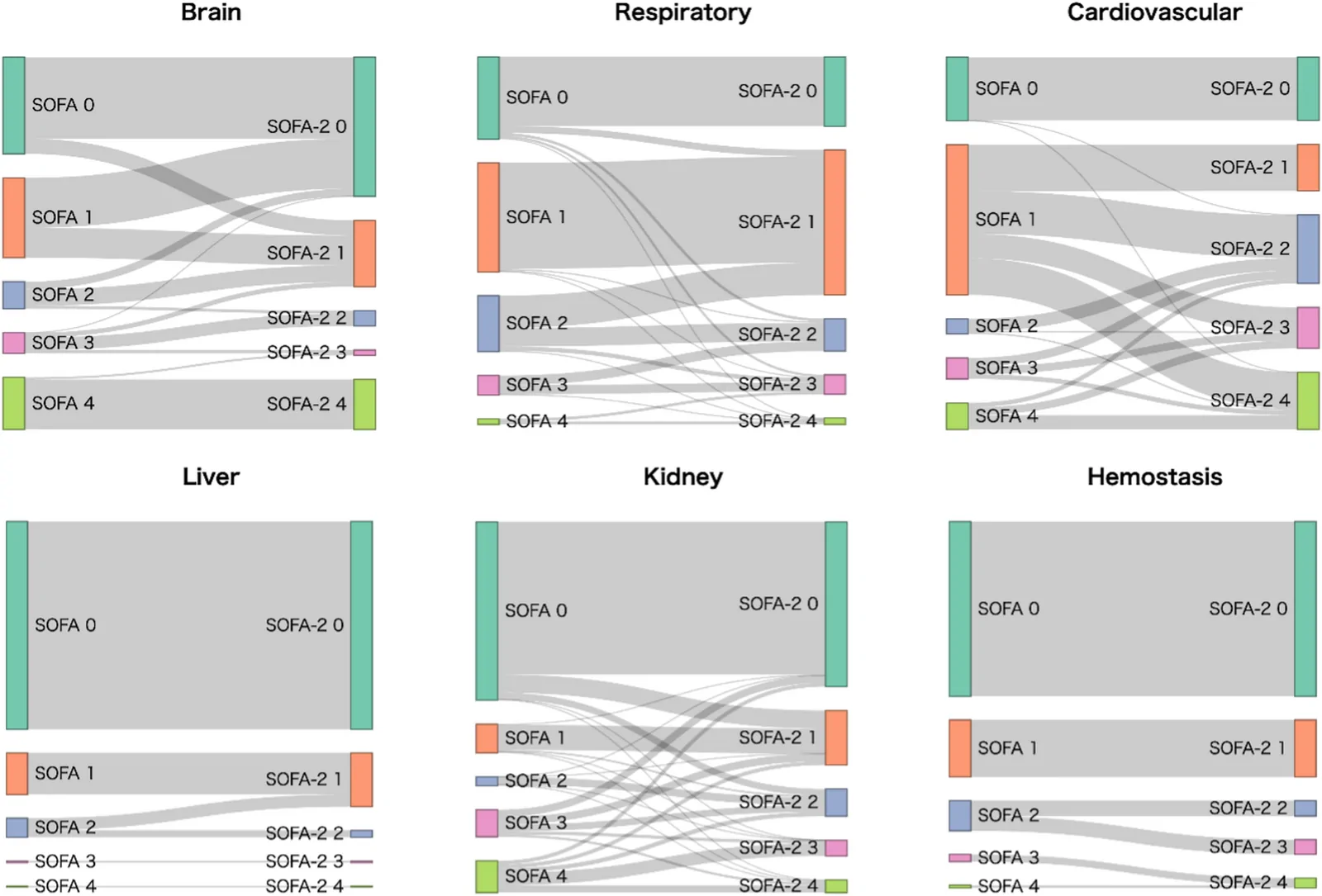
Reclassification of organ subscores between Day-1 SOFA and SOFA-2. Sankey diagrams illustrate patient-level reclassification based on day-1 organ subscores. For each organ system (Brain, Respiratory, Cardiovascular, Liver, Kidney, and Hemostasis), transitions in subscore categories (0–4) from conventional SOFA (left) to SOFA-2 (right) are shown. The width of each flow is proportional to the number of patients undergoing the corresponding transition

Figure 1 shows subscore-level reclassification patterns from SOFA to SOFA-2. For the brain subscore, most transitions were toward higher SOFA-2 subscores, with a shift from 0 to 1 also observed. Redistribution was observed within the lower score range (0–1), including shift of ≥ 2 points toward higher categories. For the respiratory subscore, transitions were concentrated within the 2–4 range, including upward changes following threshold revision. Increases of ≥ 2 points were also present. For the cardiovascular subscore, the most frequent SOFA subscore was 1, and higher subscores (2–4) were more frequent under SOFA-2. For liver and hemostasis, reclassification corresponded to threshold changes. The kidney subscore showed the most complex pattern, with substantial bidirectional reclassification, with both increases and decreases in assigned subscores under SOFA-2.

**Fig. 2:**
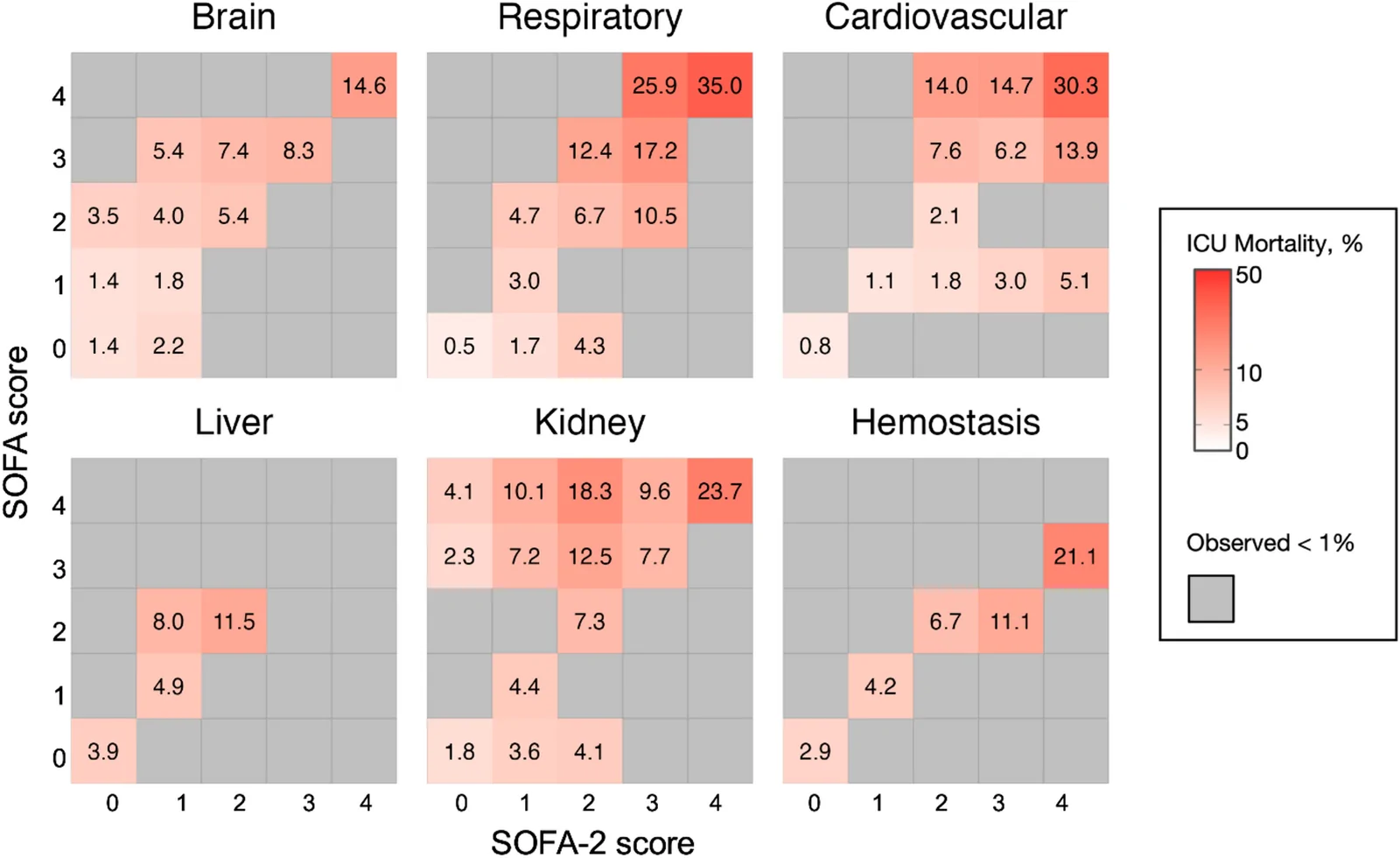
Subscore-level reclassification heat maps between Day-1 SOFA and SOFA-2 with ICU mortality. Results are based on Day 1 organ subscores. For each subscore (Brain, Respiratory, Cardiovascular, Liver, Kidney, and Hemostasis), heat maps display ICU mortality (%) for each combination of SOFA score (y-axis) and SOFA-2 score (x-axis) subscores (0–4). Values within cells indicate ICU mortality (%) and color intensity reflects the magnitude of mortality. Cells shaded in gray indicate score combinations observed in < 1% of the cohort

Figure 2 shows subscore-specific heat maps of ICU mortality across combinations of SOFA (y-axis) and SOFA-2 (x-axis) subscores. Mortality increased across the higher combinations of SOFA and SOFA-2 subscores. After fixing the SOFA-2 subscore, mortality still increased with higher SOFA subscores (i.e., vertically along the y-axis), suggesting clinically meaningful heterogeneity within the same SOFA-2 category. This vertical gradient was pronounced for the respiratory subscore at SOFA-2 subscores 2–4, for the cardiovascular subscore at SOFA-2 subscores 2–4, and for the kidney subscore at SOFA-2 subscores 1–2, where changes in observed mortality across SOFA categories were large. Similar reclassification patterns and mortality gradients were observed in the hourly analyses (Supplementary Figs. 2–3).

**Fig. 3:**
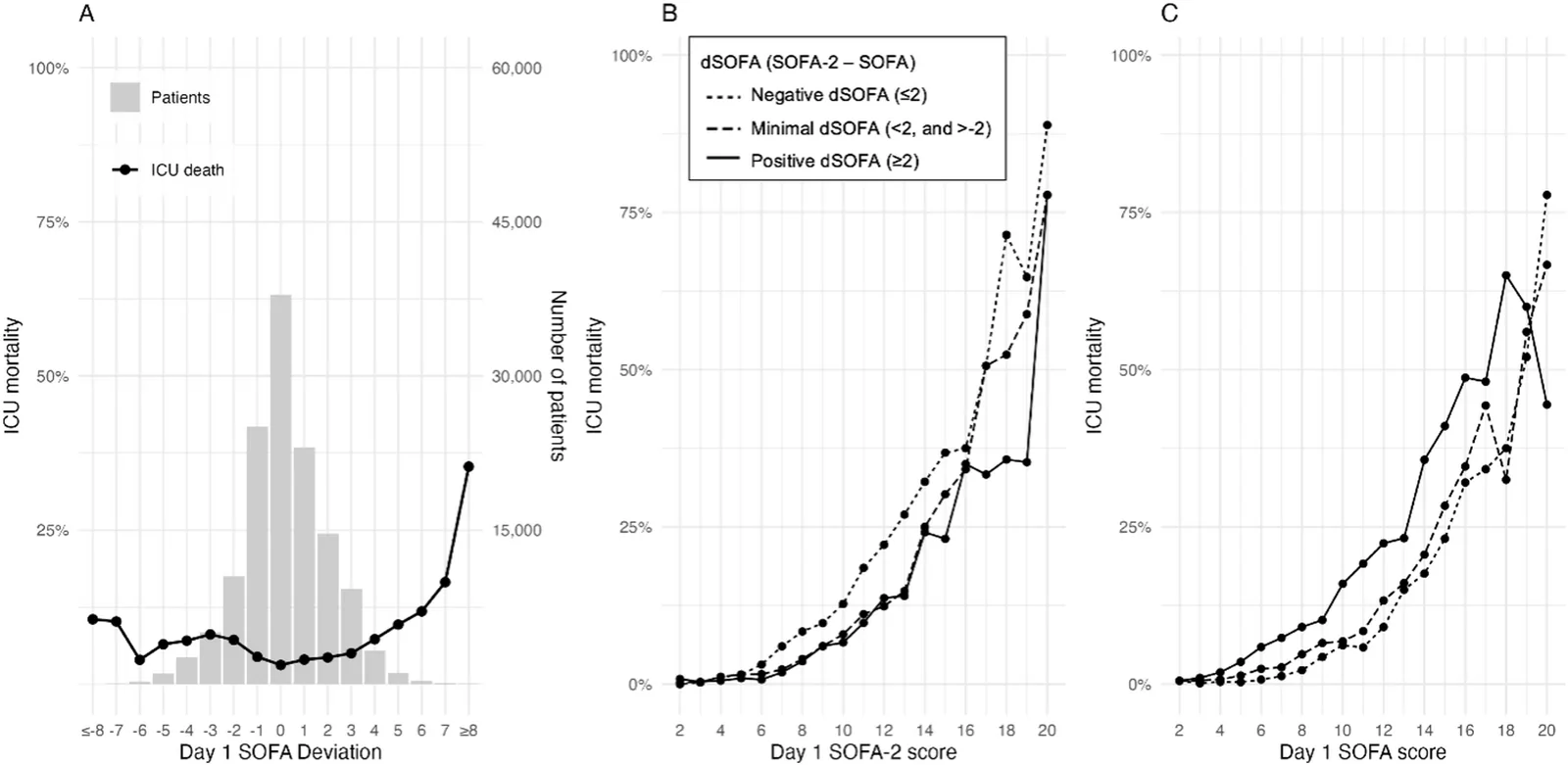
Distribution of Day-1 SOFA deviation and ICU mortality according to SOFA and SOFA-2 severity.Panel A shows the distribution of Day-1 SOFA deviation (dSOFA), defined as Day-1 SOFA-2 minus Day-1 SOFA (dSOFA = SOFA-2 − SOFA). Gray bars indicate the number of hourly observations (right y-axis), and the black line indicates observed ICU mortality (left y-axis). Panel B shows ICU mortality across Day-1 SOFA-2 scores among patients matched across dSOFA categories on the SOFA-2 score. Panel C shows ICU mortality across hourly SOFA scores among patients matched across dSOFA categories on the original SOFA score. dSOFA categories were defined as negative dSOFA (dSOFA ≤ − 2), minimal dSOFA (− 2 < dSOFA < 2), and positive dSOFA (dSOFA ≥ 2). ICU mortality represents the observed proportion of ICU deaths at each corresponding SOFA-2 or SOFA score

Overall discordance between SOFA and SOFA-2 was quantified using dSOFA (Figs. 3 and Supplementary Fig. 6). The conceptual framework for the two exact-matching analyses, conditioning separately on SOFA-2 and conventional SOFA, is illustrated using a DAG in Supplementary Fig. 4. We performed two parallel primary exact-matching analyses across the three dSOFA categories, matching separately on Day-1 SOFA-2 and Day-1 SOFA total scores, yielding 47,676 and 40,746 patients in each analysis (Table 2, Supplementary Fig. 1, and Supplementary Fig. 5). After exact matching on Day-1 SOFA-2, the three dSOFA groups had identical mean SOFA-2 scores by design (7.43 in all groups), while SOFA differed (10.18 Negative dSOFA; 7.41 Minimal dSOFA; 4.75 Positive dSOFA; *P* < 0.001). Negative dSOFA patients were older, had higher APACHE III scores, and more frequently received invasive mechanical ventilation and vasoactive therapies, Positive dSOFA patients had lower APACHE III scores and less frequent use of vasoactive agents, and more frequent renal replacement therapy. Conversely, after exact matching on Day-1 SOFA, the three groups had identical SOFA score distributions, whereas SOFA-2 scores increased from the Negative to the Positive dSOFA group. The overall gradients in severity-related characteristics and organ-support therapies across the dSOFA categories were also broadly reversed compared with those in the SOFA-2–matched cohort.

**Table 2:** Patient characteristics and outcomes after exact matching on Day-1 SOFA-2 and SOFA, stratified by SOFA Deviation (dSOFA) category

	SOFA-2 Matching					SOFA Matching			
	Negative dSOFA (n=15898)	Minimal dSOFA (n=15898)		Positive dSOFA (n=15898)	P-value	Negative dSOFA (n=13582)	Minimal dSOFA (n=13582)	Positive dSOFA (n=13582)	P-value
Age, years, mean (sd)	68.4 (15.9)	67.5 (16.1)		66.5 (17.1)	<0.001	65.9 (17.4)	67.6 (16.1)	68.2 (16.4)	<0.001
Female, n (%)	6071 (38.2)	6104 (38.4)		6346 (39.9)	0.003	5481 (40.4)	5088 (37.5)	4959 (36.5)	<0.001
BMI, kg/m2, mean (sd)	22.3 (4.6)	22.8 (10.3)		23.0 (4.4)	<0.001	22.4 (4.4)	22.6 (4.4)	22.7 (4.5)	<0.001
Readmission, n (%)	1250 (8.0)	941 (5.9)		873 (5.5)	<0.001	865 (6.4)	800 (5.9)	887 (6.5)	0.077
Elective Surgery, n (%)	2113 (13.3)	2836 (17.8)		1661 (10.4)	<0.001	1906 (14.0)	2379 (17.5)	1488 (11.0)	<0.001
APACHE III, pts, mean (sd)	111.6 (39.5)	100.1 (37.8)		89.0 (36.2)	<0.001	85.4 (35.6)	99.3 (34.9)	112.3 (34.8)	<0.001
CCI, pts, mean (sd)	4.4 (3.2)	4.3 (3.1)		3.8 (2.9)	<0.001	4.0 (3.1)	4.3 (3.1)	4.1 (3.1)	<0.001
Treatment, n (%)									
Nasal High Flow	434 (2.7)		435 (2.7)	520 (3.3)	0.004	208 (1.5)	354 (2.6)	604 (4.4)	<0.001
NIPPV	377 (2.4)		413 (2.6)	635 (4.0)	<0.001	226 (1.7)	380 (2.8)	650 (4.8)	<0.001
IMV	8269 (52.0)		6356 (40.0)	4263 (26.8)	<0.001	3887 (28.6)	5389 (39.7)	6323 (46.6)	<0.001
VV-ECMO	13 (0.1)		15 (0.1)	14 (0.1)	0.931	3 (0.0)	11 (0.1)	24 (0.2)	<0.001
Epinephrine	380 (2.4)		234 (1.5)	54 (0.3)	<0.001	113 (0.8)	167 (1.2)	164 (1.2)	0.002
Vasopressin	1121 (7.1)		667 (4.2)	302 (1.9)	<0.001	337 (2.5)	487 (3.6)	481 (3.5)	<0.001
Norepinephrine	7956 (50.0)		3587 (22.6)	756 (4.8)	<0.001	3536 (26.0)	2754 (20.3)	1420 (10.5)	<0.001
Dopamine	2168 (13.6)		2070 (13.0)	344 (2.2)	<0.001	994 (7.3)	1635 (12.0)	580 (4.3)	<0.001
Dobutamine	1827 (11.5)		1817 (11.4)	434 (2.7)	<0.001	758 (5.6)	1385 (10.2)	679 (5.0)	<0.001
IABP	140 (0.9)		167 (1.1)	220 (1.4)	<0.001	49 (0.4)	111 (0.8)	270 (2.0)	<0.001
Microaxial flow pump	15 (0.1)		29 (0.2)	15 (0.1)	0.036	4 (0.0)	11 (0.1)	32 (0.2)	<0.001
VA-ECMO	119 (0.7)		147 (0.9)	145 (0.9)	0.166	31 (0.2)	86 (0.6)	289 (2.1)	<0.001
RRT	581 (3.7)		875 (5.5)	1180 (7.4)	<0.001	189 (1.4)	671 (4.9)	2010 (14.8)	<0.001
SOFA score, pts, mean (sd)	10.2 (3.5)		7.40 (3.5)	4.7 (3.3)	<0.001	7.3 (3.0)	7.3 (3.0)	7.3 (3.0)	1
SOFA-2 score, pts, mean (sd)	7.41 (3.4)		7.41 (3.4)	7.41 (3.4)	1	4.7 (3.0)	7.3 (3.1)	10.0 (3.2)	<0.001
Outcomes									
ICU death	1423 (9.0)		909 (5.7)	796 (5.0)	<0.001	462 (3.4)	683 (5.0)	1291 (9.5)	<0.001
Hospital death, n (%)	2272 (14.6)		1472 (9.5)	1320 (8.5)	<0.001	917 (6.9)	1185 (8.9)	1952 (14.6)	<0.001
Length of ICU Stay, days, mean (sd)	8.2 (11.3)		6.9 (11.9)	6.7 (10.2)	<0.001	5.9 (9.1)	6.6 (9.2)	8.8 (13.0)	<0.001
Length of Hospital Stay, days, mean (sd)	33.1 (46.0)		29.2 (29.1)	27.2 (35.5)	0.064	27.3 (43.2)	32.2 (34.1)	35.1 (46.1)	0.045

SOFA Deviation (dSOFA) was defined as the difference between Day-1 SOFA2 and Day-1 SOFA scores (Day-1 SOFA2 minus Day-1 SOFA). Patients were stratified as Negative dSOFA (≤ −2), Minimal dSOFA (> −2 and < 2), and Positive dSOFA (≥ 2). Patients were matched across the three groups on Day-1 SOFA-2 score and SOFA score. Categorical variables are presented as number (%); variables not labeled as n (%) are numerical and are presented as mean (SD). Abbreviations: APACHE III, Acute Physiology and Chronic Health Evaluation III; BMI, Body Mass Index; CCI, Charlson Comorbidity Index; IABP, Intra-Aorta Balloon Pumping; IMV, Invasive Mechanical Ventilation; NIPPV, Non-Invasive Positive Pressure Ventilation; RRT, Renal Replacement Therapy; VV-ECMO, Veno-Venous Extracorporeal Membrane Oxygenation; VA-ECMO, Veno-Arterial ExtraCorporeal Membrane Oxygenation

**Fig. 4:**
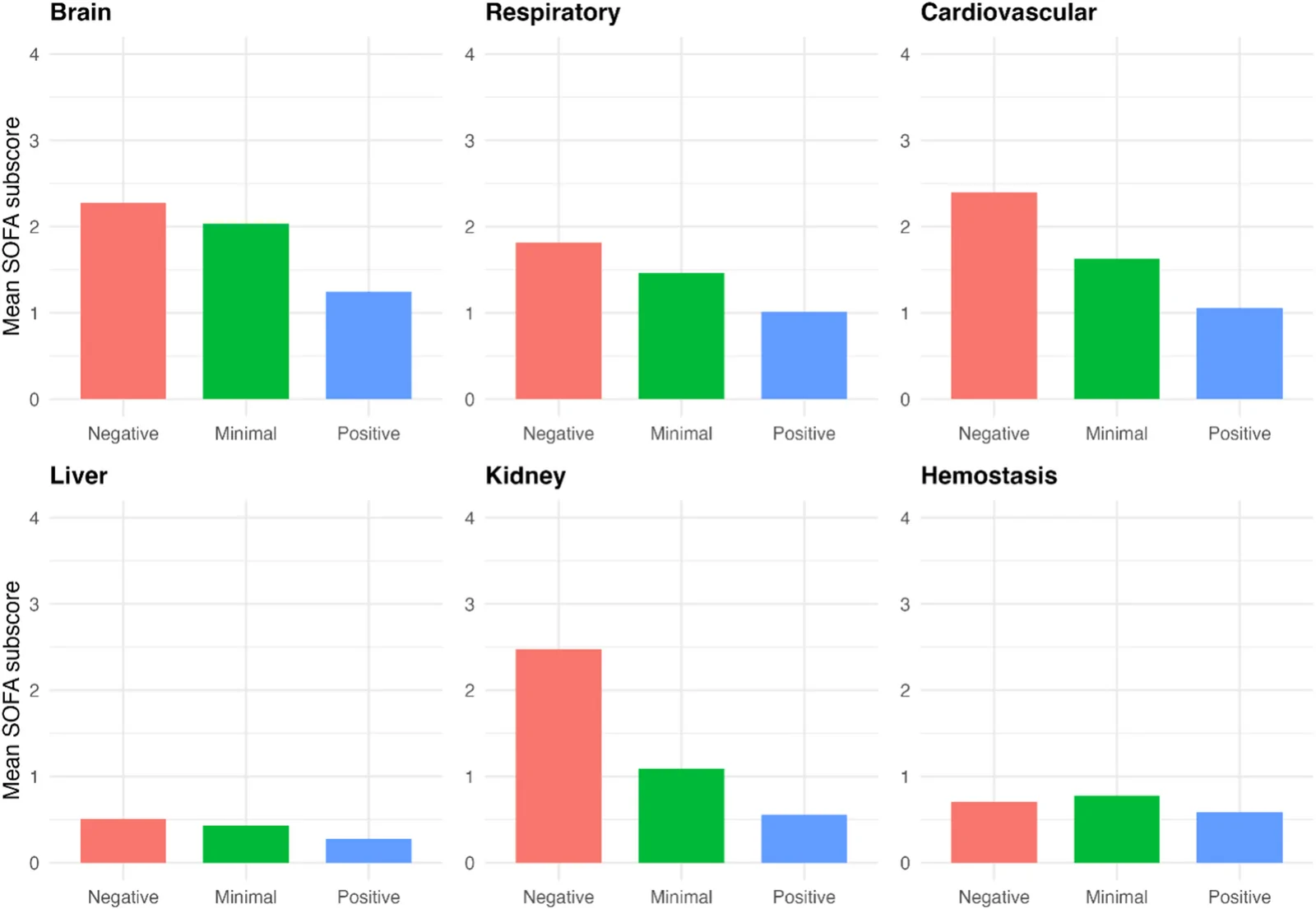
Organ-specific SOFA subscores across SOFA deviation categories. Mean baseline (Day 1) organ subscores (brain, respiratory, cardiovascular, liver, kidney, and hemostasis) stratified by SOFA deviation (dSOFA) category. dSOFA deviation was defined as Day-1 SOFA-2 minus Day-1 SOFA (dSOFA = SOFA-2 − SOFA). Categories were defined as Positive deviation (dSOFA ≥ 2), Minimal deviation (− 2 < dSOFA < 2), and Negative deviation (dSOFA ≤ − 2). Bars represent mean SOFA subscores (range 0–4) within each deviation category. Color coding indicates deviation category: red = Negative deviation, green = Minimal deviation, and blue = Positive deviation

In Figs. 3B and Supplementary Fig. 6B, after matching on SOFA-2, ICU mortality differed across deviation categories. Mortality was highest in the Negative dSOFA group, intermediate in the Minimal dSOFA group, and lowest in the Positive dSOFA group. This separation between deviation categories widened as SOFA-2 severity increased. In the parallel analysis matching on SOFA, ICU mortality tended to be higher in the Positive dSOFA group, which had higher SOFA-2 scores within the same SOFA strata (Fig. 3C and Supplementary Fig. 6C). Figure 4 and Supplementary Fig. 7 summarize Day 1 SOFA organ subscores across dSOFA categories within the cohort matched on SOFA-2 score. With the exception of hemostasis, subscores were highest in the Negative dSOFA group, followed by the Minimal dSOFA group, and lowest in the Positive dSOFA group. This gradient was observed for the kidney and cardiovascular subscores. The distribution of kidney subscores differed across Day-1 SOFA categories. In Supplementary Fig. 7, the proportion of patients in each kidney SOFA category varied across dSOFA groups.

Sensitivity analyses showed broadly consistent findings. In the inverse probability weighting analysis using multinomial propensity scores for dSOFA categories, absolute mean differences were below 0.1 after weighting (Supplementary Fig. 9), and mortality differences across deviation categories were consistent with the SOFA-2–matched analysis (Supplementary Fig. 8A, B). In the sepsis subgroup, estimates were more variable at higher score levels, but the overall pattern remained consistent with the SOFA-2–matched analysis (Supplementary Fig. 8C, D).

## Discussion

This study examined the transition from SOFA to SOFA-2 and yielded several important findings. Sankey diagrams demonstrated systematic upward shifts toward higher severity categories, particularly in the brain, cardiovascular, and respiratory subscores. Heat map analyses showed that mortality increased with higher SOFA subscores when the SOFA-2 subscore was fixed. Furthermore, discordance between SOFA and SOFA-2 was associated with clinically meaningful outcome differences even after matching on SOFA and SOFA-2.

Our findings suggest that SOFA-2 represents more than a simple adjustment of score thresholds; rather, it introduces a systematic reclassification of organ dysfunction severity. This reclassification primarily affected the neurological, cardiovascular, and respiratory subscores, reflecting updates that incorporate contemporary ICU practices, including mechanical support therapies such as extracorporeal membrane oxygenation, the inclusion of vasoactive agents such as vasopressin, and modifications to neurological assessment [7–9, 21, 22]. Assigning higher scores to organ support is intuitively consistent with clinical severity. However, prior work has shown that assigning the maximum score to extracorporeal support does not necessarily improve mortality discrimination [10]. Given that SOFA is designed to classify organ dysfunction rather than predict mortality, improvements in discrimination are not required for its validity. Our Sankey analysis indicates that patients reclassified to the highest category represent a small proportion and may have limited impact on overall discrimination. The effect of this reclassification may be greater in settings enriched with advanced organ support, such as ECMO centers. At the same time, incorporating extracorporeal support into scoring may introduce double counting of severity and obscure changes in patient status during support. The performance of SOFA and SOFA-2 for longitudinal assessment remains to be established.

In contrast, the kidney subscore demonstrated a more complex pattern with bidirectional reclassification. This pattern reflects attempts to capture renal dysfunction using detailed definitions but also highlights unresolved limitations. Upward shifts likely reflect urine output criteria and the inclusion of renal replacement therapy, whereas downward shifts may occur after discontinuation of renal support. Because renal replacement therapy is assigned the maximum subscore while ongoing, improvement in underlying renal function may be obscured during treatment [23]. In addition, current scoring approaches may not fully capture the timing and degree of renal recovery following discontinuation of renal replacement therapy [24]. In addition, reliance on creatinine has been questioned [25]. In our analysis, heat maps demonstrated prognostic heterogeneity within identical SOFA-2 categories. Furthermore, although more detailed definitions may improve discrimination, they also increase computational complexity [26]. Overall, assessment of renal dysfunction remains challenging [27], and limitations persist even in the revised SOFA-2 framework [28].

We next examined the clinical implications of score discordance using SOFA deviation. After matching on SOFA-2, mortality was higher in the Negative dSOFA group, whereas after matching on SOFA, mortality was higher in the Positive dSOFA group. These findings indicate that patients classified at the same severity level by one scoring system may remain clinically heterogeneous according to the other scoring system and with respect to outcome. Similar findings were reported in an external validation study, in which patients with SOFA-2 lower than SOFA had the highest ICU mortality (9.8%), compared with 7.8% in upward reclassification and 4.3% in unchanged scores [18]. Within our two matched cohorts, the distributions of illness severity, organ-support therapies, and other clinical characteristics across dSOFA categories were broadly reversed according to the score used for matching. Differences in treatment use, particularly renal replacement therapy and vasoactive medications, likely contributed to discordance through their effects on the renal and cardiovascular subscores. These domains include overlapping components, such as dialysis, urine output, and creatinine for renal function, and vasoactive agents and mechanical support for cardiovascular function. The findings therefore suggest that SOFA and SOFA-2 capture overlapping but nonidentical aspects of organ dysfunction, rather than indicating that either score is uniformly more informative. SOFA-2 nevertheless has important advantages, including the incorporation of clinically relevant contemporary variables and improved predictive performance, which may be further enhanced in specific settings [29].

### Interpretation and implications

Our findings may have practical implications for the clinical interpretation of organ dysfunction scores. Prior validation studies have reported higher prognostic discrimination for SOFA-2 than for conventional SOFA, supporting its clinical relevance as an updated severity scoring system. However, higher prognostic discrimination does not necessarily imply that conventional SOFA should be replaced entirely or that SOFA-2 should be preferentially used in all clinical settings. Instead, the two scores may provide complementary information regarding patient heterogeneity that is not fully represented by either scoring system alone. The DAG illustrated that most parameters contributing to SOFA and SOFA-2 overlap. Conditioning on one score would therefore be expected to account for much of the information represented by the other. Nevertheless, clinically meaningful differences in the alternate score, patient characteristics, and mortality remained after either matching strategy. This pattern suggests that the residual heterogeneity may arise not only from differences in the variables included in each score, but also from differences in their scoring algorithms, including thresholds, weighting, and the treatment of organ-support therapies. Discordance between SOFA and SOFA-2 may therefore identify patients whose severity is not fully captured by one scoring system alone. Such discordance may provide complementary information during clinical assessment, but whether it leads to specific actionable decisions remains uncertain. Our study did not evaluate whether SOFA-2–based reclassification improves prognostic calibration, changes bedside decisions, or improves patient outcomes. Future studies should investigate whether discordance between the scores can be used for risk stratification or clinical decision support.

Another possible interpretation is that SOFA and SOFA-2 differ in how they operationalize severity. A key algorithmic distinction is that SOFA-2 assigns the maximum subscore to certain advanced organ-support modalities. This approach is clinically intuitive and may have contributed to the improved mortality discrimination reported for SOFA-2. However, potential limitations of assigning the maximum score to patients receiving advanced organ support have been reported in several previous studies [30–32]. Iwasaki et al. described this limitation as a “ceiling effect” [30], whereas Kumaido et al. reported a case in which SOFA and SOFA-2 appeared to provide complementary information during ongoing extracorporeal and renal support [23]. Accordingly, the relative contribution of each score may vary across phases of critical illness, including initial resuscitation, escalation of organ-support therapy, and recovery. This variation may partly explain the discordance observed between the two scores. Further studies are needed to clarify how assigning maximum subscores for advanced organ support affects longitudinal assessment and clinical interpretation.

### Strengths and limitations

This study has several strengths. First, the use of high-resolution ICU data enabled detailed analysis of score transitions and their association with clinical outcomes. The OneICU database records vital signs at one-minute intervals and includes detailed records of therapies and laboratory measurements, allowing granular evaluation of organ dysfunction trajectories [33, 34]. In addition, all variables required to calculate SOFA and SOFA-2 were available in the database, and both scores could be assigned for all eligible admissions using observed data and prespecified score-assignment rules. Second, the multicenter design within a single healthcare system allowed evaluation across multiple ICU settings in Japan.

However, several limitations should be considered. First, some component variables were not clinically measured in individual patients. In accordance with the prespecified score-assignment rules, observations without an available measurement were assigned a score of zero until the first measurement was recorded. Therefore, although SOFA and SOFA-2 could be calculated for all eligible admissions, comparisons at the component level should be interpreted with caution. Second, the study was conducted in 15 Japanese hospitals during a period that included the COVID-19 pandemic, which may limit generalizability across regions and time periods [35]. The relatively high Charlson Comorbidity Index may also affect interpretation because chronic organ dysfunction, including chronic kidney disease, can elevate SOFA and SOFA-2 subscores independently of acute organ failure [27, 36]. Because admission dates were anonymized, pandemic-related effects could not be assessed. Third, we did not evaluate the cost-effectiveness of increased scoring complexity, including the balance between the burden of score calculation and potential improvements in predictive performance [26]. Future studies should address whether severity scoring systems should evolve toward more complex physiological models or prioritize simplicity and bedside usability. Fourth, our score-based approach may be inherently reductionist because summary scores cannot fully represent complex states of organ dysfunction. Although SOFA was developed to describe organ dysfunction, it was refined partly according to its association with mortality, creating a conceptual tension between its descriptive and prognostic roles. Moreover, an increase in SOFA score is now widely used in the operational definition of sepsis. Our findings should therefore be interpreted as score-based associations rather than as a comprehensive or causal representation of organ dysfunction.

## Conclusion

In this study, we evaluated patient-level changes in organ subscores and total severity scores when transitioning from SOFA to SOFA-2 and examined their association with clinical outcomes. SOFA-2 induced systematic reclassification of organ dysfunction, and reciprocal exact-matching analyses showed that patients assigned the same severity by one scoring system remained heterogeneous according to the other. Simultaneous interpretation of SOFA and SOFA-2 may provide complementary information for severity assessment. Future work should determine whether organ dysfunction assessment tools should evolve toward more comprehensive physiological models or maintain simpler frameworks optimized for bedside use.

## Supplementary Information

Below is the link to the electronic supplementary material.

Supplementary Material 1.

## Data Availability

The datasets generated and/or analyzed during the current study are not available since the dataset was obtained from MediCU through a formal request/approval process as above but is available from the corresponding author on reasonable request.

## Abbreviations

APACHE: Acute Physiology and Chronic Health Evaluation
BMI: Body Mass Index
CCI: Charlson Comorbidity Index
DAG: directed acyclic graph
dSOFA: SOFA deviation (SOFA-2 minus SOFA)
ICU: Intensive Care Unit
IABP: Intra-Aortic Balloon Pumping
NPPV: Non-Invasive Positive Pressure Ventilation
RRT: Renal Replacement Therapy
SD: Standard Deviation
SOFA: Sequential Organ Failure Assessment
SOFA-2: Revised Sequential Organ Failure Assessment score
STROBE: Strengthening the Reporting of Observational Studies in Epidemiology
VV-ECMO: Veno-Venous Extracorporeal Membrane Oxygenation
VA-ECMO: Veno-Arterial Extracorporeal Membrane Oxygenation
