# Supplementary material for: Patient-level reclassification and discordance between SOFA and SOFA-2: a multicenter ICU study
Source: Crit Care. 2026 Aug 11;30:425. doi: 10.1186/s13054-026-06241-0 (PMC13483513; doi:10.1186/s13054-026-06241-0)
Supplement: Supplementary file 1 [file 13054_2026_6241_MOESM1_ESM.docx]

**Supplementary Material**

**Supplementary Methods.** General definitions and data processing

**Supplementary Figure 1.** Study flow diagram for cohort selection and data inclusion.

**Supplementary figure 2.** Reclassification of organ subscores between Hourly SOFA and SOFA-2.

**Supplementary figure 3.** Subscore-level reclassification heat maps between Hourly SOFA and SOFA-2 with ICU mortality.

**Supplementary Figure 4.** Distribution of SOFA-2 scores before and after matching by dSOFA category.

**Supplementary Figure 5.** Distribution of Hourly SOFA deviation and ICU mortality according to SOFA-2 severity.

**Supplementary Figure 6.** Distribution of Day-1 SOFA subscores by SOFA deviation category.

**Supplementary figure 7.** ICU mortality across SOFA and SOFA-2 scores according to dSOFA category using SOFA-score matching, inverse probability weighting, and a sepsis subgroup analysis.

**Supplementary figure 8.** Covariate balance before and after inverse probability weighting according to dSOFA category.

**Supplementary Table 1.** Proportion of Patients with Unmeasured Variables Required for SOFA and SOFA-2 Score Calculation.

**Supplementary Methods. General definitions and data processing**

- SOFA and SOFA-2 scores were calculated in accordance with the original and updated definitions, respectively. ICU admission was defined as time zero (time 0), and the observation period was divided into consecutive 1-hour intervals (time window indices), which served as the unit of analysis.
- The OneICU database automatically extracts routinely recorded ICU data from electronic intensive care information systems, including vital signs, laboratory results, organ support, and administered treatments. Therefore, data availability reflected routine clinical documentation and measurement frequency rather than manual abstraction.
- Source data recorded at finer temporal resolutions, including minute-level vital signs and treatment records, were summarized into each 1-hour interval according to prespecified rules. For mean arterial pressure, PaO₂/FiO₂ ratio, SpO₂/FiO₂ ratio, and GCS, the lowest recorded value within each time window was used, after applying the component-specific rules described below. For total bilirubin, creatinine, and potassium, the highest recorded value was used. For platelet count, pH, and bicarbonate, the lowest recorded value was used.
- For time windows without available measurements immediately after ICU admission, the corresponding component subscores were assigned as zero until the first observation was recorded. For mean arterial pressure, SpO₂, and laboratory variables (PaO₂, platelet count, total bilirubin, creatinine, bicarbonate, pH, and potassium), values were forward-filled until the next available measurement.
- Absence of a record for organ support or treatment was interpreted as absence of that support or treatment during the corresponding time window.
- For vasoactive and other continuously administered drugs, the maximum recorded dose within each time window was used.
- SOFA and SOFA-2 scores calculated at each time window were defined as hourly SOFA and hourly SOFA-2, respectively. When multiple measurements were available within a time window, the worst value (corresponding to the highest subscore) was used.
- Day-1 SOFA and Day-1 SOFA-2 scores were defined as the sum of the worst organ-specific subscores observed across all time windows within the first 24 hours after ICU admission.
- To improve transparency regarding unmeasured physiologic data, we added a supplementary table showing the proportion of patients without recorded vital sign measurements (Supplementary Table 1).
- Additional component-specific rules are described below.

**Brain**

- For the neurological component, the Glasgow Coma Scale (GCS) total score was used for both SOFA and SOFA-2, whereas the GCS motor score was used only for SOFA-2.
- In both SOFA and SOFA-2, when patients were under sedation, the best pre-sedation GCS score was used. If patients were intubated, the GCS motor score was used in place of the total score when appropriate.
- For SOFA, if patients were intubated but not sedated, the observed GCS total score was used without modification.
- For SOFA-2, delirium treatment was defined as the presence of a positive Confusion Assessment Method for the ICU (CAM-ICU) or a Richmond Agitation–Sedation Scale (RASS) score ≥ +1, in combination with the administration of any sedative agent.

**Respiratory**

- For SOFA, only invasive mechanical ventilation was classified as advanced respiratory support, and respiratory scoring was based on the PaO₂/FiO₂ ratio.
- For SOFA-2, invasive mechanical ventilation, non-invasive positive pressure ventilation, and nasal high-flow oxygen therapy were classified as advanced respiratory support. The PaO₂/FiO₂ ratio was used when available; the SpO₂/FiO₂ ratio was used only when no PaO₂/FiO₂ measurement was available during the assessment period.

**Cardiovascular**

- For SOFA, cardiovascular scoring was based on mean arterial pressure and the use of norepinephrine, dopamine, dobutamine, and epinephrine, according to the original SOFA definition.
- For SOFA-2, cardiovascular scoring was based on the updated SOFA-2 criteria. In addition to norepinephrine, dopamine, dobutamine, and epinephrine, phosphodiesterase III inhibitors (e.g., milrinone), β-adrenergic stimulants (e.g., isoproterenol), and vasoconstrictive agents (e.g., vasopressin, phenylephrine, and methylene blue) were classified as “any other vasopressor or inotrope.”

**Kidney**

- Urine output criteria were applied based on predefined time windows. For SOFA-2, 6-hour and 12-hour urine output thresholds were evaluated only when the observation period exceeded the corresponding duration. For SOFA and SOFA-2 24-hour urine output criteria, values were calculated as the average urine output rate from the observation point up to the corresponding time window.
- The criterion of “non-renal use” in SOFA-2 was not applied due to difficulty in operational definition within the database.
- For non-dialysis periods after intermittent renal replacement therapy, score assignment was based on prior dialysis exposure and fulfillment of RRT indication criteria, defined as creatinine level greater than 1.2 mg/dL (>110 μmol/L) or oliguria (<0.3 mL/kg/h) for more than 6 hours, plus at least one of serum potassium ≥6.0 mmol/L or metabolic acidosis with pH ≤7.20 and serum bicarbonate ≤12 mmol/L.

**Other components**

- No additional modifications were applied to the coagulation (hemostasis), or liver components.

**Abbreviations**

SOFA, Sequential Organ Failure Assessment; SOFA-2, updated Sequential Organ Failure Assessment score; GCS, Glasgow Coma Scale; RASS, Richmond Agitation–Sedation Scale; CAM-ICU, Confusion Assessment Method for the Intensive Care Unit; SpO₂, peripheral oxygen saturation; PaO₂, partial pressure of arterial oxygen; NHF, nasal high-flow oxygen therapy; NIPPV, non-invasive positive pressure ventilation; ECMO, extracorporeal membrane oxygenation; RRT, renal replacement therapy.

**Supplementary Figure 1. Study flow diagram for cohort selection and data inclusion.** SOFA deviation (dSOFA) was defined as SOFA-2 minus SOFA (dSOFA = SOFA-2 − SOFA) and categorized as Positive dSOFA (≥2), Minimal dSOFA (−2 < dSOFA < 2), and Negative dSOFA (≤−2).

**Supplementary figure 2. Reclassification of organ subscores between Hourly SOFA and SOFA-2**. Sankey diagrams show reclassification based on hourly organ subscores. For each subscore (Brain, Respiratory, Cardiovascular, Liver, Kidney, and Hemostasis), transitions in subscore categories (0–4) from SOFA (left) to SOFA-2 (right) are depicted. The width of each flow is proportional to the number of patients in the corresponding transition.

**Supplementary figure 3. Subscore-level reclassification heat maps between Hourly SOFA and SOFA-2 with ICU mortality.** Results are based on Hourly 1 organ subscores. For each subscore (Brain, Respiratory, Cardiovascular, Liver, Kidney, and Hemostasis), heat maps display ICU mortality (%) for each combination of SOFA score (y-axis) and SOFA-2 score (x-axis) subscores (0–4). Values within cells indicate ICU mortality (%) and color intensity reflects the magnitude of mortality. Cells shaded in gray indicate score combinations observed in <1% of the cohort.

**Supplementary figure 4. Conceptual Directed Acyclic Graph (DAG) of SOFA and SOFA-2 components in relation to ICU mortality.** Blue boxes represent organ dysfunction domains, white boxes represent observed component variables, the green box represents underlying severity, and the dark blue box represents the outcome. Variables shown in the area labeled “SOFA-2 only” are incorporated only in SOFA-2, whereas those in the area labeled “SOFA and SOFA-2” are incorporated in both scoring systems. The graph was constructed using DAGitty (<https://www.dagitty.net/>). Abbreviations: GCS, Glasgow Coma Scale; ICU, intensive care unit; IMV, invasive mechanical ventilation; MCS, mechanical circulatory support; NHF, nasal high-flow oxygen therapy; NIPPV, noninvasive positive pressure ventilation; RRT, renal replacement therapy; SOFA, Sequential Organ Failure Assessment; VV-ECMO, venovenous extracorporeal membrane oxygenation.

**Supplementary Figure 5. Distribution of SOFA-2 and SOFA scores before and after exact matching across dSOFA categories.** Panels A and C show the distributions of SOFA-2 scores before and after exact matching on the SOFA-2 total score, respectively. Panels B and D show the distributions of SOFA scores before and after exact matching on the SOFA total score, respectively. Exact matching was performed separately across the three SOFA deviation (dSOFA) categories, resulting in identical distributions of the corresponding matching score among the groups. dSOFA was defined as SOFA-2 minus SOFA (dSOFA = SOFA-2 − SOFA) and categorized as negative deviation (dSOFA ≤ −2), minimal deviation (−2 < dSOFA < 2), or positive deviation (dSOFA ≥ 2). Abbreviations: dSOFA, difference between SOFA-2 and SOFA; SOFA, Sequential Organ Failure Assessment.

**Supplementary Figure 6. Distribution of hourly SOFA deviation and ICU mortality according to SOFA and SOFA-2 severity.** Panel A shows the distribution of hourly SOFA deviation (dSOFA), defined as hourly SOFA-2 minus hourly SOFA (dSOFA = SOFA-2 − SOFA). Gray bars indicate the number of hourly observations (right y-axis), and the black line indicates observed ICU mortality (left y-axis). Panel B shows ICU mortality across hourly SOFA-2 scores among patients matched across dSOFA categories on the SOFA-2 score. Panel C shows ICU mortality across hourly SOFA scores among patients matched across dSOFA categories on the original SOFA score. dSOFA categories were defined as negative dSOFA (dSOFA ≤ −2), minimal dSOFA (−2 < dSOFA < 2), and positive dSOFA (dSOFA ≥ 2). ICU mortality represents the observed proportion of ICU deaths at each corresponding SOFA-2 or SOFA score.

**Supplementary Figure 7. Distribution of Day-1 SOFA subscores by SOFA deviation category.** Stacked panels show the distribution of Day-1 SOFA-2 organ-specific subscores (0–4) for Brain, Respiratory, Cardiovascular, Liver, Kidney, and Hemostasis, stratified by SOFA deviation category. SOFA deviation (dSOFA) was defined as Day-1 SOFA-2 minus Day-1 SOFA (dSOFA = SOFA-2 − SOFA). Categories were defined as Negative deviation (dSOFA ≤ −2), Minimal deviation (−2 < dSOFA < 2), and Positive deviation (dSOFA ≥ 2). For each organ and deviation category, bars represent the proportion (%) of patients at each SOFA-2 subscore level such that the proportions across subscores sum to 100% within each category.

**Supplementary figure 8. ICU mortality across SOFA and SOFA-2 scores according to dSOFA category using inverse probability weighting, and a sepsis subgroup analysis of SOFA-2 matching.** dSOFA was defined as SOFA-2 minus SOFA and categorized as Negative deviation (dSOFA ≤ −2), Minimal deviation (−2 < dSOFA < 2), and Positive deviation (dSOFA ≥ 2). Panels A–B: Inverse probability weighting (IPW) analyses using propensity scores for dSOFA category. Panels C–D: Sepsis subgroup analyses matched on SOFA-2 score. Panels A and C show Day-1 analyses; Panels B and D show hourly analyses. Abbreviations: SOFA, Sequential Organ Failure Assessment; dSOFA, SOFA deviation.

**Supplementary figure 9. Covariate balance before and after inverse probability weighting according to dSOFA category.** Absolute standardized mean differences (SMDs) for baseline characteristics before (red) and after (blue) inverse probability weighting (IPW) for the average treatment effect (ATE). dSOFA was defined as SOFA-2 minus SOFA and categorized as Negative deviation (dSOFA ≤ −2), Minimal deviation (−2 < dSOFA < 2), and Positive deviation (dSOFA ≥ 2). Points represent the maximum absolute SMD across pairwise comparisons among the three dSOFA categories. The dashed vertical line indicates an SMD of 0.10, commonly used as a threshold for acceptable covariate balance.

**Supplementary Table 1. Proportion of Patients with Unmeasured Variables Required for SOFA and SOFA-2 Score Calculation**

| **Domain** | **SOFA unmeasured n (%)** | **SOFA-2 unmeasured n (%)** |
| --- | --- | --- |
| Respiration | 40,576 (30.2%) | 354 (0.26%) |
| Cardiovascular | 79 (0.1%) | 79 (0.1%) |
| Hemostasis | 2,907 (2.2%) | 2,907 (2.2%) |
| Brain | 17,418 (12.9%) | 3,728 (2.8%) |
| Liver | 6,524 (4.9%) | 6,524 (4.9%) |
| Kidney | 449 (0.3%) | 446 (0.3%) |

Values represent the number and percentage of patients for whom all variables required to calculate the corresponding organ subscore were unmeasured during the first 24 hours after ICU admission. Unmeasured status was defined as the absence of all variables required for calculation of a given organ subscore during this period.
